# Epigastric hernia as a rare manifestation of a bile duct cyst and gallbladder cancer: A first case report

**DOI:** 10.1016/j.ijscr.2020.02.058

**Published:** 2020-02-29

**Authors:** Mauricio Alves Ribeiro, Gabriel Lorente Mitsumoto, André Soares Gallo, Luiz Arnaldo Szutan

**Affiliations:** a School of Medicine, Santa Casa de Sao Paulo School of Medical Sciences, Sao Paulo, SP, Brazil; b Liver and Portal Hypertension Group of Surgery Department, Santa Casa de Sao Paulo School of Medical Sciences, Sao Paulo, SP, Brazil

**Keywords:** Bile duct cyst, Epigastric hernia, Gallbladder cancer, Hernia, Ventral

## Abstract

**Introduction:**

Elective repair epigastric hernia is a frequent minor surgical procedure. In most cases the hernial content is pre-peritoneal fat.

**Presentation of case:**

We report the case of a patient with epigastric hernia containing part of a bile duct cyst.

**Discussion:**

Bile duct cysts are often asymptomatic, but when symptoms are present they may include intermittent, recurrent epigastric or right hypochondrial pain; abdominal tenderness; fever and mild jaundice.

**Conclusion:**

The presence of a bile duct cyst within a hernia is a very rare finding, especially making the diagnosis through an epigastric hernia. This case report is the first of a bile duct cyst within an epigastric hernia.

## Highlights

• Gallbladder cancer is asymptomatic in the early stages, but when symptoms are present, they are similar to biliary colic or chronic cholecystitis.
• Epigastric hernia is usually asymptomatic too, with localized pain being the main symptom when present.
• Abdominal ultrasound showed a giant cyst and cholelithiasis.
• The presence of a bile duct cyst within a hernia is a very rare finding, especially making the diagnosis through an epigastric hernia.

## 1 Introduction

Bile duct cysts may be asymptomatic, with initial recognition being incidental from imaging studies for unrelated reasons. When symptoms are present, the most common are intermittent recurrent epigastric or right hypochondrial pain, abdominal tenderness, fever and mild jaundice [1]. Nausea, vomiting and anorexia may also be present. Cancer of the gallbladder is also asymptomatic in the early stages, but when symptoms are present, they are similar to biliary colic or chronic cholecystitis.

Epigastric hernia is usually asymptomatic too, with localized pain being the main symptom when present. In obese patients the diagnosis can be difficult, but in thin patients a bulge may be seen or palpated [2]. This is the first case of a bile duct cyst and a gallbladder cancer being discovered following diagnosis of an epigastric hernia, with the hernia content containing part of the cyst. This work is reported in line with the SCARE criteria [3].

## 2 Case report

**Fig. 1:**
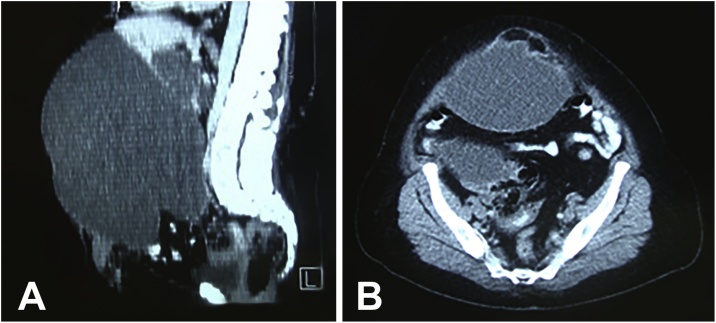
A: CT showed a giant bile duct cyst. B: CT confirmed that epigastric hernia content contained part of the cyst.

A 53-year-old woman was referred to our hospital with a diagnosis of epigastric hernia. She had had an enlarged, bulging epigastric region and abdominal pain in the right hypochondrium and epigastrium for a year. Abdominal ultrasound showed a giant cyst and cholelithiasis. Computed tomography (CT) showed a cyst of 33 × 23 × 30 cm with content estimated at 12,000 cm^3^ (12 liters) and confirmed that the epigastric hernia content contained part of the cyst (Fig. 1A, B).

**Fig. 2:**
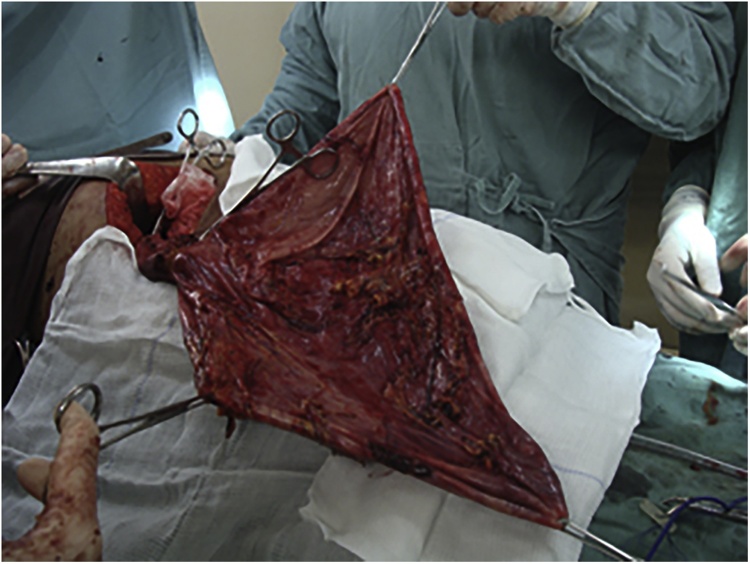
Resection of the bile duct cyst.

The patient was subjected to resection of the cyst, cholecystectomy and Mayo repair of the epigastric hernia (Fig. 2). A supraumbilical median laparotomy was performed, assisted with monopolar electric scalpel. Intraoperative pathological analysis confirmed the diagnosis of a biliary cyst and gallbladder adenocarcinoma T1b. Thus, a limited 3-cm wedge resection of the gallbladder bed and lymph node dissection were performed. Skin closure was performed by primary suture with separated stitches. The patient is asymptomatic at 6 years follow-up, free of the disease and without signs of recurrence of the hernia.

## 3 Discussion

There is still controversy as to how exactly epigastric hernia occurs. Both the vascular lacunae hypothesis and the forceful contraction hypothesis are accepted as possible explanations for the etiology [4]. Most of these hernias are made up of pre-peritoneal fat only. Furthermore, these hernias, unlike other types of hernia, do not always possess a peritoneal sac. Also, apart from the omentum, intestinal contents such as the stomach, small intestine, colon, Meckel´s diverticulum and even gallbladder are rarely seen in the sac [[5], [6], [7]].

The most common symptoms of bile duct cysts are abdominal pain, fever and jaundice, but their diagnosis is often is an incidental finding. The incidental finding of an early stage gallbladder neoplasm allows, in most cases, a better prognosis [8]. A suture or mesh repair should be performed in small and large defects respectively [9]. In this case, although there was a large defect, the resection of the cyst allowed a Mayo repair without tension.

In a review of the literature, we searched the PubMed database for articles published up to July 2018 using the terms “hernia AND bile duct cyst”, “hernia AND gallbladder cancer” and “epigastric hernia AND cancer” and identified 161 articles. The titles and the abstracts of the articles were evaluated, but none of them reported something similar to our case.

The presence of a bile duct cyst within a hernia is a very rare finding, especially making the diagnosis through an epigastric hernia. This case report is the first of a bile duct cyst within an epigastric hernia.

## Funding

There was no source of funding for this research. Any costs were covered by the authors.

## Ethical approval

The Institutional Ethics Committee approval number is 2.819.549. Ethics committee approved this study with waiver of the signed consent term, since patient having her case reported lost follow-up after a few years, and it is no longer possible to contact her by phone. In addition, given the nature of the study, we will use only the information provided in the patient's medical record, the data will be analyzed in a way that will not make it possible for her to be identified, and there will be no discrimination team that could compromise his or her privacy or integrity.

## Consent

Written informed consent was not obtained from the patient. The head of our medical team has taken responsibility that exhaustive attempts have been made to contact the family and that the paper has been sufficiently anonymised not to cause harm to the patient or their family. A copy of a signed document stating this is availble for review by the Editor-in-Chief of this journal on request.

## Author contribution

Mauricio Alves Ribeiro: conception and design, acquisition of data. Literature research development of data set and write manuscript.

Gabriel Lorente Mitsumoto: acquisition of data. Literature research development of data set and write manuscript.

André Soares Gallo: acquisition of data.

Luiz Arnaldo Szutan: conception and design. Final approval of the version to be published.

## Registration of research studies

Our manuscript is a case report not a research.

## Guarantor

Luiz Arnaldo Szutan, MD, PhD. Associate professor in the Surgery Department, at Santa Casa de Sao Paulo School of Medical Sciences, School of Medicine, São Paulo, Brazil. Head of the Liver and Portal Hypertension Group of Irmandade da Santa Casa de Misericórdia de São Paulo, São Paulo Brazil.

## Provenance and peer review

Not commissioned, externally peer-reviewed.

## Declaration of Competing Interest

The authors declare that there are no conflicts of interest.
